# Prediction of cytotoxicity of heavy metals adsorbed on nano-TiO_2_ with periodic table descriptors using machine learning approaches

**DOI:** 10.3762/bjnano.14.77

**Published:** 2023-09-12

**Authors:** Joyita Roy, Souvik Pore, Kunal Roy

**Affiliations:** 1 Drug Theoretics and Cheminformatics Laboratory, Department of Pharmaceutical Technology, Jadavpur University, Kolkata, 700032, India

**Keywords:** heavy metals, HK-2 cell, ML algorithm, periodic table descriptors, QSAR

## Abstract

Nanoparticles with their unique features have attracted researchers over the past decades. Heavy metals, upon release and emission, may interact with different environmental components, which may lead to co-exposure to living organisms. Nanoscale titanium dioxide (nano-TiO_2_) can adsorb heavy metals. The current idea is that nanoparticles (NPs) may act as carriers and facilitate the entry of heavy metals into organisms. Thus, the present study reports nanoscale quantitative structure–activity relationship (nano-QSAR) models, which are based on an ensemble learning approach, for predicting the cytotoxicity of heavy metals adsorbed on nano-TiO_2_ to human renal cortex proximal tubule epithelial (HK-2) cells. The ensemble learning approach implements gradient boosting and bagging algorithms; that is, random forest, AdaBoost, Gradient Boost, and Extreme Gradient Boost were constructed and utilized to establish statistically significant relationships between the structural properties of NPs and the cause of cytotoxicity. To demonstrate the predictive ability of the developed nano-QSAR models, simple periodic table descriptors requiring low computational resources were utilized. The nano-QSAR models generated good *R*^2^ values (0.99–0.89), *Q*^2^ values (0.64–0.77), and *Q*^2^*F*_1_ values (0.99–0.71). Thus, the present work manifests that ML in conjunction with periodic table descriptors can be used to explore the features and predict unknown compounds with similar properties.

## Introduction

Nanoparticles (NPs) have gained much attention due to their widespread applications in different areas, and they are continually designed to yield certain desired properties [1]. With the uninterrupted development of new NPs, engineered nanoparticles in the form of metal oxide nanoparticles are becoming a new area of research. Metal oxides have been used in different industries, and the market is developing rapidly [2]. According to a recent analysis, approximately 1300 consumer products containing NPs were marketed in 2012. As a common metal oxide nanoparticle material, nanoscale titanium dioxide (nano-TiO_2_) has been evaluated for diverse applications. TiO_2_ has been shown to be a promising material for practical applications because it is highly photoreactive, inexpensive, non-toxic, chemically and biologically inert, and photostable. Also, nano-TiO_2_ exhibits high specific surface area and anti-corrosion and photocatalytic properties [3]. It absorbs UV radiation and shows self-cleaning ability. Nanoparticles have a susceptibility to adsorb other substances to form a mixture leading to a shift of toxicity to living organisms [4]. Hence, many studies have reported cytotoxic characteristics of TiO_2_ [5–6].

Some NPs are fatal to living cells, and their cytotoxicity may inhibit cell growth cycles, leading to death of organisms. Considering this fact, the cytotoxicity of TiO_2_ in combination with other pollutants has been evaluated. TiO_2_ is the most commonly manufactured nanoparticle material. It is assumed that because of the considerably high exposure TiO_2_ NPs may enter the food chain. Because of current industrialization processes, organisms are also exposed to heavy metal pollutants [7]. Emitted NPs may interact with the pollutants, and this may subsequently lead to bioaccumulation. The contamination of water and soil with heavy metals has increased with anthropogenic and industrial activities [8–9]. TiO_2_ NPs commonly co-exist with different heavy metals as they are released from wastewater treatment facilities to freshwater bodies, affecting the mode of action and the fate of the contamination. Studies have reported the ability of TiO_2_ NPs to adsorb heavy metals and to increase their transport rate into hosts, increasing their concentration in the cell. Hu et al. [10] investigated the joint effect of TiO_2_ NPs and humic acid (HA) on Cd^2+^ bioaccumulation in zebrafish. In another study, Yang et al. [11] showed that TiO_2_ NPs increased the accumulation of Cd^2+^ in the ciliate *Tetrahymen thermophila*. Further, Tan et al. [12] showed increased uptake and retention of Cd^2+^ and Zn^2+^ adsorbed on TiO_2_ NPs in *Daphnia magna*. Heavy metal contamination affects plant growth and indirectly affects human health via the food chain. Heavy metals have become an important factor limiting crop yields and, thus, threatening food security. Therefore, to improve crop yields, heavy metals need to be removed.

The toxicity of single-substance NPs has been tested extensively; however, the combination of single-substance NPs with other NPs or metals may cause co-exposure effects on living organisms. The extensive use of heavy metals in areas such as medicine and agriculture increased the negative impact of heavy metals on environment and living organisms, raising the need for risk assessment. Unlike other pollutants, heavy metals do not decompose, leading to bioaccumulation and biological hazards [13]. Heavy metals enter the human body through the consumption of fish and plants [14]. To date, heavy metals are removed through various methods. Among all methods available for removing heavy metals and toxic pollutants from waters, adsorption is the most widely used. Therefore, the joint organismal toxicity should be assessed.

Recently, nanoscale quantitative structure–activity relationship (nano-QSAR) models have been successfully applied to investigate the toxicity of NPs. QSAR models for predicting the biological activity of 48 fullerene derivatives [15], 51 manufactured nanoparticles with varying core metals, coatings, and surface attachments [16], and 80 surface-modified multiwall carbon nanotubes have been reported. Another approach, namely nano-read-across (nano-RA) [17], has been used to determine the cytotoxicity of unknown nanomaterials based on structure similarities with known substances. Materials with similar structures are likely to produce similar toxicity through comparable mechanisms. The development of machine learning (ML) approaches, such as artificial neural networks (ANNs), decision trees, logistic regression (LR), support vector machines (SVM), Naïve Bayes (NB), random forest (RF), and *k*-nearest neighbor (*k*-NN), can be used to construct models that simulate complex relationships [18] and make predictions based on training data.

Using ensemble learning (EL) [19] methods, one can determine the relationship between the response and the predictor as well as solve regression problems. Additionally, such methods overcome problems with weak predictors and can be used to reduce the overfitting of the training data by averaging and incorporating multiple models. Ensemble learning is established with multiple algorithms and is divided into bagging and boosting algorithms. The boosting algorithm is an iterative algorithm that uses a weak model to build a strong model. Both bagging and boosting improve the prediction accuracy of weaker learners. A boosting algorithm combines many models linearly, with each new model depending on the previous one. In the bagging algorithm, replica data sets are generated that minimize prediction variance in machine learning. An iterative algorithm performs a series of repeated steps to gradually improve the model’s performance or to optimize a specific parameter. The algorithms continue to update the model’s parameters based on the training data until a certain stopping criterion is met, such as reaching an optimal solution, or a predefined number of steps are completed. This process is performed during the training of the model, where the model learns from the data by adjusting its parameters to minimize a specific cost or error function. These algorithms play a crucial role in training machine learning models and are fundamental to many optimization and learning techniques. Fine-tuning the model parameters through iterations helps to improve the model’s performance and makes it more suitable for making accurate predictions for new, unseen data.

The boosting algorithm is an ensemble method that works sequentially by adding predictors to an ensemble, each one correcting its predecessors. In the boosting algorithm, at first, an initial model is developed with the dataset and then the algorithm tries to adjust the model parameters and again develops a model that tries to correct or minimize errors present in the previous model. This process is repeated until a satisfactory model is obtained or the error function is significantly optimized. Through this process, we get a strong learner or model from several weak learners or models by sequentially minimizing the error present in the predecessor models. Here, the weak model represents the models that are developed at an initial stage and contain a significant amount of error. The strong model is indicated by the final model, which contains a significantly low level of error and is able to predict new unknown data more accurately. Bagging (or bootstrap aggregating) is an ensemble method that generates a number of bootstrap datasets by a method called random sampling with replacement, and each dataset is used to train the models separately. The final prediction is obtained by averaging the outcome of each model (for regression models) or by majority voting (for classification models).

The objective of the present study was to construct EL-based regression models (RF, Gradient Boost, Extreme Gradient Boost, and AdaBoost) with periodic table descriptors for predicting the cytotoxicity, in terms of cell viability, of eight heavy metals adsorbed on nano-TiO_2_. Also, the best algorithm showing the most contributing features responsible for the toxicity to HK-2 (human kidney 2) cell has been determined. To the best knowledge of the authors, this is the first work on ML models using periodic table descriptors to successfully demonstrate the high potential of the proposed modeling approaches.

## Methods and Materials

### Dataset

The dataset was collected from previously published literature [20]. A mixture of nano-TiO_2_ powders was added to HK-2 cells in Hyclone DMEM medium supplemented with 10% fetal bovine serum (FBS) and 100 mg penicillin/streptomycin and maintained at 37 °C in the presence of 5% carbon dioxide. Nine concentrations of heavy metal salts were added to a constant amount of nano-TiO_2_ (25 µmol/L). The details of heavy metal concentrations are given in Table 1.

**Table 1:** Different concentrations of heavy metal salt samples in µmol/L.

Sample	CdCl_2_	ZnCl_2_	CuSO_4_	NiCl_2_	Pb (NO_3_)_2_	MnCl_2_	SbCl_3_	CoCl_2_

1	10	60	30	100	100	100	5	10
2	20	90	60	200	200	200	10	20
3	30	120	90	300	300	300	15	30
4	40	150	120	400	400	400	20	40
5	50	180	150	500	500	500	25	50
6	60	210	180	600	600	600	30	60
7	70	240	210	700	700	700	35	70
8	80	270	240	800	800	800	40	80
9	90	300	270	900	900	900	45	90

HK-2 cells were utilized to determine the toxicity in this study using cell viability as the endpoint. HK-2 cells are a sensitive model for examining renal cytotoxicity. They grow in monolayers and are suitable for studying the proximal tubular toxicity of a variety of compounds [21]. The main advantage of HK-2 cells is that they retain the basic morphological and functional properties of proximal tubular epithelial cells [22]. Cell viability was measured by using Equation 1:


[1] $$S=\frac{A_{\exp}-A_{\text{blank}}}{A_{\text{control}}-A_{\text{blank}}}.$$

Here, *S* stands for cell survival rate, *A*_exp_ is the absorbance value of the experimental group, *A*_control_ is the absorbance value of the control group, and *A*_blank_ is the absorbance value of the blank control group.

### Descriptor calculation

Based on the characteristics of metals, we used easily calculable periodic table descriptors. Simple molecular information was generated time-effectively and cost-effectively. The previously used descriptors by Kar et al. [23] are the metal electronegativity (χ), the sum of metal electronegativity for an individual metal oxide (∑χ), the sum of metal electronegativity for an individual metal oxide divided by the number of oxygen atoms present in that metal oxide (∑χ/NO), the number of metal atoms (*N*_Metal_), the number of oxygen atoms (*N*_Oxygen_), the charge of the metal cation in a given oxide (χox), and the molecular weight (MW). These descriptors are termed “first-generation periodic table descriptors”. The newly introduced sixteen descriptors are denoted as “second-generation periodic table descriptors” [24]. The computed descriptors for all metals are reported in the Excel file in Supporting Information File 1. In addition to being computationally less demanding, periodic table descriptors are size-independent.

### Splitting of data set and hyperparameter tuning

The dataset was split into training and test sets before building the model. The training set was mainly used to fit the model, and the test set was used to measure the generalization ability of the developed model. Theoretically speaking, the dataset was divided based on a sorted response-based approach using the in-house dataset division tool (https://dtclab.webs.com/software-tools). In this study, the size ratio was set at 3:1 (training set/test set) for dataset division.

In almost any ML algorithm, different models are trained for a dataset and the best-performing model is selected. However, there may be room for improvement, and hyperparameter tuning can significantly improve the model. Here, the optimal values of the hyperparameters of the models were obtained with the GridSearchCV algorithm using the hyperparameter optimizer tool (https://sites.google.com/jadavpuruniversity.in/dtc-lab-software/home/machine-learning-model-development-guis?pli=1). GridSearchCV tests all combinations of values in the dictionary and evaluates the model using the cross-validation method for each combination. Therefore, we choose the hyperparameter combination with the best average MAE results from the validation sets.

### Feature selection with random forest

The goal of feature selection techniques is to find the best set of features that allows one to build optimized models. Feature selection using RF is an embedded method. Embedded methods combine the benefits of filter and wrapper techniques. These methods encompass the interaction of features while maintaining reasonable computational cost. In embedded methods, each iteration of the model training process is taken care of, and a few features that contribute the most to the training process are carefully extracted. More precisely, it is measured how much impurity is reduced on averaging (weighted average) through each tree nodes with the selected features. Here, each node is equivalent to the number of training samples associated with it. Through the RF algorithm, we have selected the most contributing eight periodic table descriptors, namely “conc”, ”∑χ”, “atomic radius”, “IP_ActivM”, “Mol_Wt”, “χ of metal”, “D3_HeteroNonMetal”, and the total number of atoms in a molecule, from a pool of 43 periodic table descriptors by using the features with the highest Gini importance [25]. The selected first eight descriptors (most contributing features) were further used for modeling using RF, AdaBoost, Gradient Boost, and Extreme Gradient Boost algorithms.

### Model development

**Figure 1:**
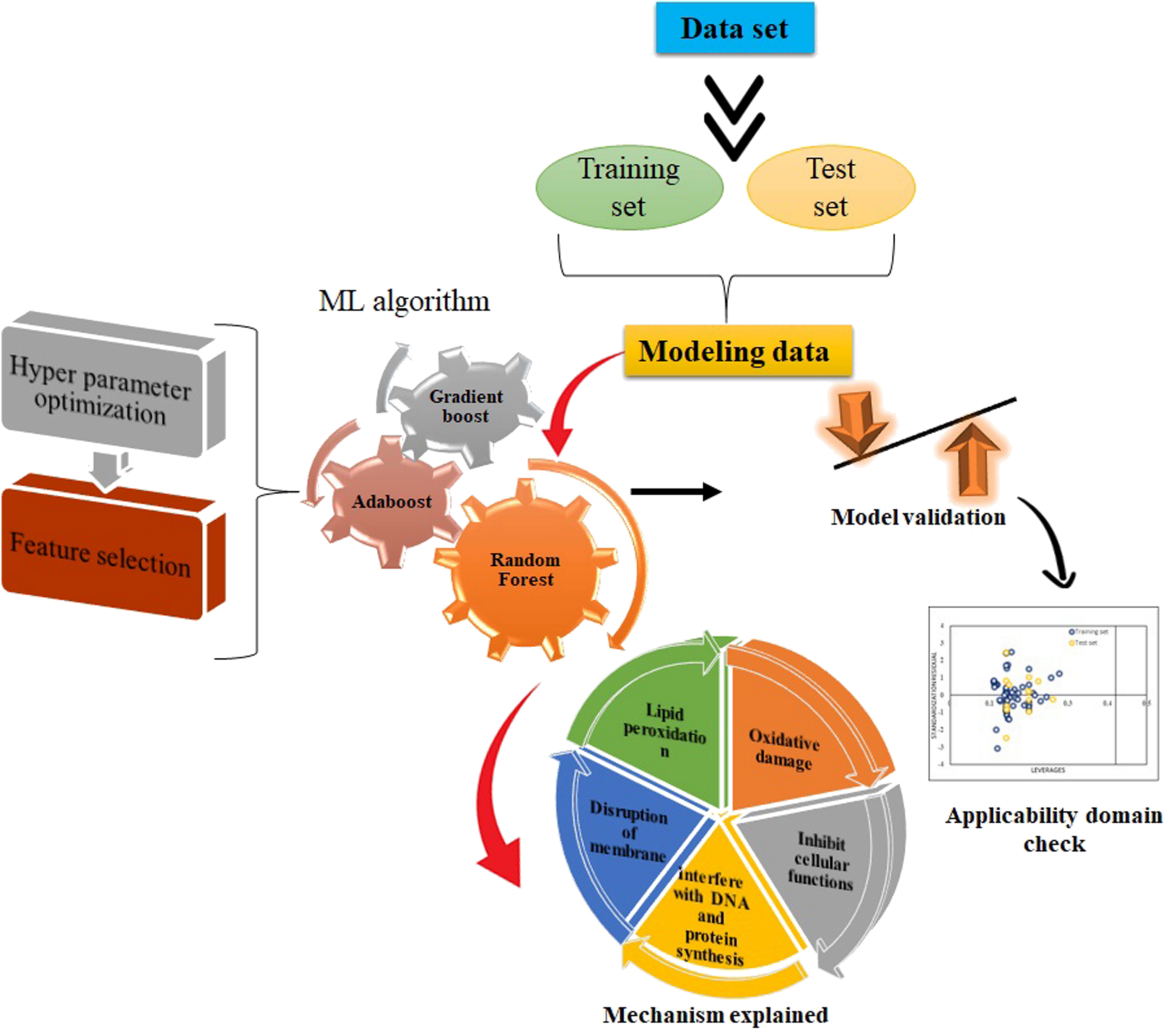
Flowchart of the present work.

This section introduces four classification models; all of them are ensemble learning models. ML is a subset of artificial intelligence where the machine learns from data and improves performance from past experiences and makes a prediction based on it [26]. In this study, along with RF, Gradient Boost, Extreme Gradient Boost, and AdaBoost were also performed. In the supervised learning approach, a model is trained on labeled datasets. Regression analysis algorithms are trained and learned from both input features and output labels. Regression analysis seeks a mapping function from the input features for a continuous output function. In this study, there is no intention to categorize the dataset, instead it is to be predicted quantitatively. Hence, the supervised regression method is selected to map the function of heavy metals and predict the cytotoxicity of these metals on HK-2 cells with periodic table descriptors. For the model development, after dataset division and feature selection, different ML algorithms are performed. The overall workflow is illustrated in Figure 1.

#### Random forest (RF)

In ensemble learning, RF is often used for its flexibility. Whether it is regression or classification, RF is a versatile learning method that can handle both. It works by building several decision trees in the training phase and generates average forecasts of various decision trees involved. In other words, it combines the results of different decision trees to make the best possible decision. Though the goal variable in classification-based issues is categorical, numerical values are present in regression. One advantage of RF is its capacity to analyze large datasets with great efficiency [27]. It can be regarded as a dimensionality reduction method since it analyzes large input data and finds all important variables. While handling RF datasets, the model emphasizes the importance of parameters, which is a highly helpful aspect [28].

#### Adaptive boosting (AdaBoost)

AdaBoost is one of the best boosting algorithms. It uses an ensemble learning method. This approach of machine learning is based on the idea of creating accurate prediction rules by combining many relatively weaker and inaccurate rules and assisting in alleviating overfitting issues. It is possible to make a smarter learner by altering the training data intelligently and constructing many submodels. It includes an unlimited amount of decision trees for input data throughout the training stage. During the creation of the first decision tree, incorrect data are highlighted inside the primary model.

The identical data serve as input for a separate model. This procedure is repeated until a specific number of base learners is generated. It uses a weighted average relying on the subsets to determine whether it should be included in the finalized model. In reality, some data may include linear predictions, and others may not. Therefore, utilizing the ensemble AdaBoost allows us to capture the nonlinear predictions and make a precise prediction for such data [29].

#### Gradient Boost (GB)

In 2002, Friedman [30] suggested an ensemble learning algorithm for both regression and classification. The GB method is associated with each repetition of the randomly chosen training data set with the fundamental model. Overfitting is inhibited by randomly subsampling the training set data; by doing so, the execution time and model accuracy are also improved. Since every repetition of the model must include small data (as a training set) the regression becomes quicker. The GB approach also requires modification or changes in a few parameters. That is, n-trees should not be too small, and the shrinkage aspect, also recognized as the learning rate, must not be kept too high [31].

#### Extreme Gradient Boost (XGBoost)

**Figure 2:**
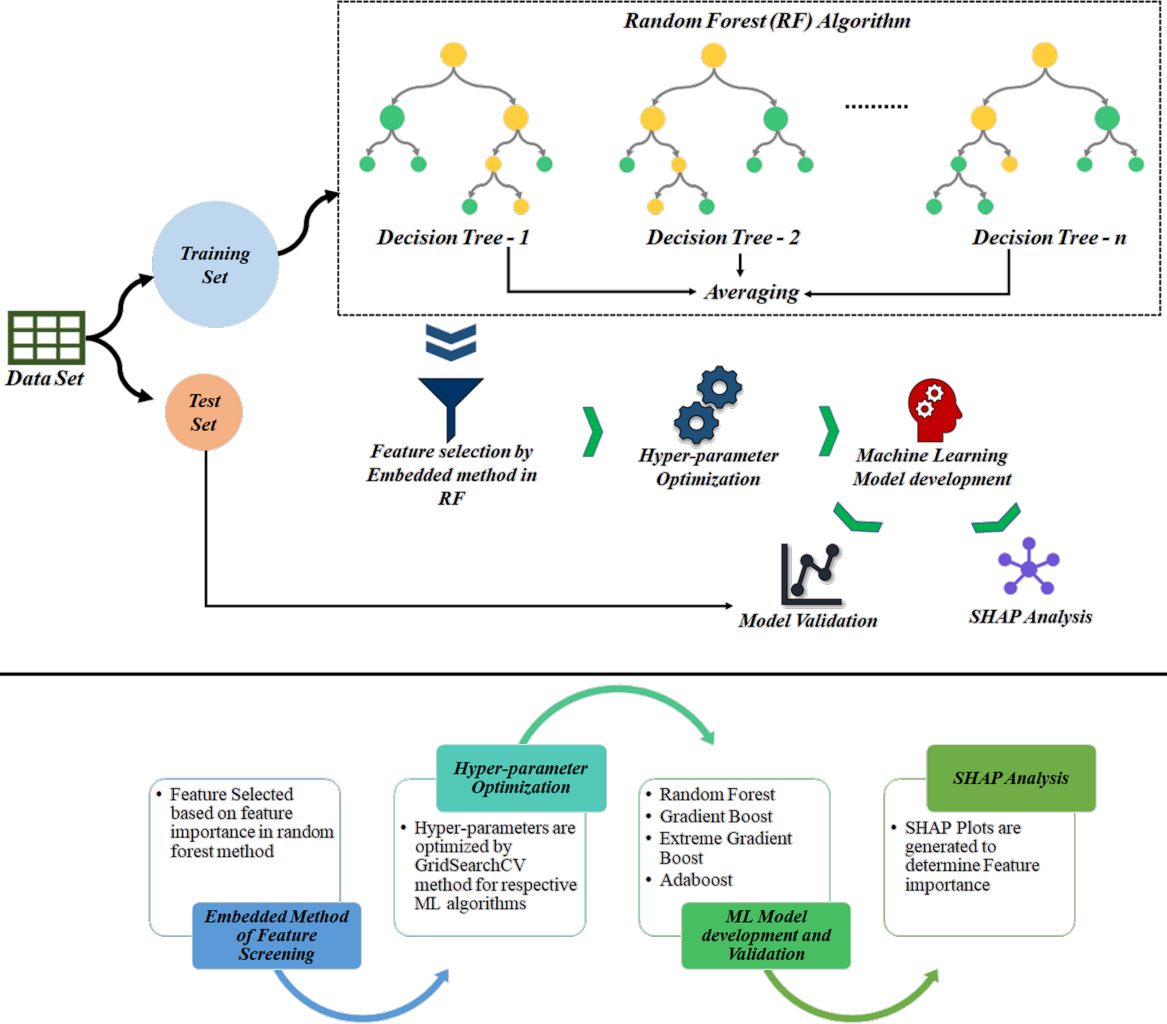
Workflow of the ML algorithm.

In a similar manner as described in [32], another ensemble ML algorithm, XGBoost of tree boosting, uses a gradient-boosting framework for efficient and scalable implementation performance. Ensemble learning uses multiple predictions that are multiple models for gradient enhancement and yields good adaptability to outliers and continuous variables. It is an efficient tool for dealing both regression and classification problems. The basic idea is to build “N” regression trees to train each subsequent tree using the residual from the previous tree. Models are built recursively until there is no improvement in the results obtained. The new models predict the residuals of the prior model and then collectively provide the final predictions [33]. The gradient descent algorithm is used to minimize the loss while adding new models. Then, these individual predictors or classifications are combined to give more strong and more precise predictions. The workflow of the ML algorithm is represented in Figure 2. Tuning can be done using the grid search method.

### SHAP analysis

The feature importance in the model was determined using the Shapley Additive exPlanation (SHAP) method, using SHAP version 0.41.0. The SHAP framework takes into account the calculation of Shapley values. These values are calculated from the average marginal contribution of each feature from all conceivable coalitions. First, the dataset is incorporated into the model, then the SHAP framework assigns a Shapley value to each feature that contributes to the corresponding output of the model. Therefore, SHAP helps to select the features based on a ranking algorithm [34]. We have selected the features having the highest Shapley values for the training set since the standard method tends to overestimate the continuous variables.

### Model validation

A reliable model should pass the threshold values for different internal and external validation metrics. Internal validation generates the generalization ability and robustness of the model. In contrast, external prediction is used to validate the model. The most common metrics to measure internal quality are the coefficient of determination (*R*^2^ and *Q*^2^_LOO_). Besides these, we have also calculated the root mean square error (RMSE) of the training set. The mean absolute error (MAE_(test)_), the root mean square error of prediction (RMSEP), *Q*^2^*F*_1_, and *Q*^2^*F*_2_ were used for external validation or for the test set. The evaluation criteria included are as follows: *Q*^2^_LOO_ and *Q*^2^*F*_1_ greater than 0.5, RMSE between 0.2 and 0.5, and the closer the value of MAE is to 0, the better [35].

### Applicability domain (AD) analysis

After building the model, the applicability domain (AD) must be considered. AD represents the domain that can be effectively predicted by the model that is based on the training set data. The samples within the domain of applicability can only explain the reliability of the predicted values. A William’s plot was used to determine the AD of the present work. The leverages were calculated using the in-house Hi_Calculator-v1 Software (https://sites.google.com/jadavpuruniversity.in/dtc-lab-software/home?pli=1). The distance between the X value of the *i*-th observation and all X values is represented by the leverage value. It generally considers 3*k*′/*N* as the critical value or the standard value (h*). Here, *k*′ represents the number of descriptors plus 1, and *N* represents the number of compounds in the training set. If the leverage value is higher than h*, the corresponding compound is outside the AD.

## Results and Discussion

**Figure 3:**
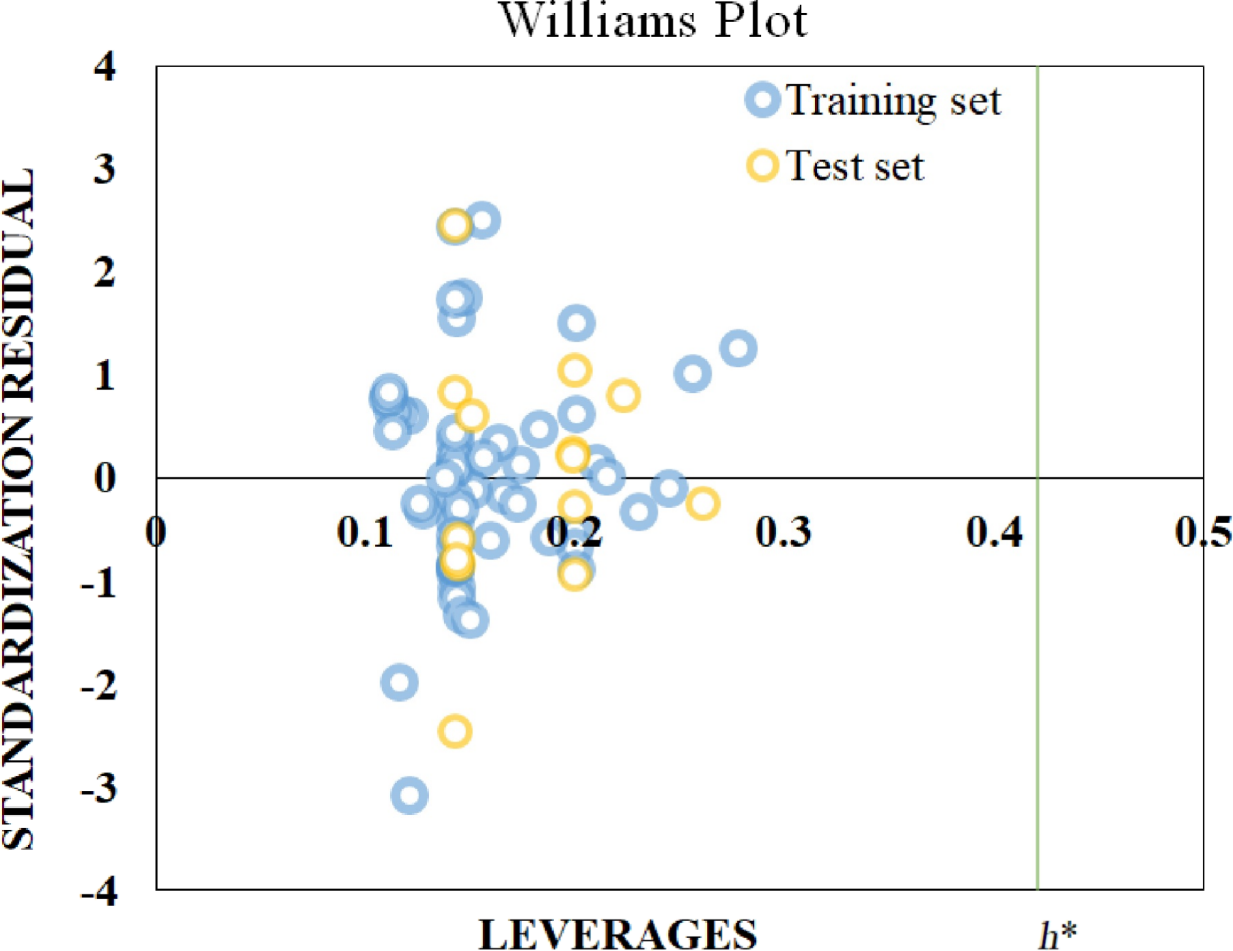
Williams plot.

In this research, we have used four ML models, namely RF, AdaBoost, Gradient Boost, and Extreme Gradient Boost to forecast the toxicity of heavy metals adsorbed on nano-TiO_2_ to HK-2 cells using periodic table descriptors (Table 2). The ML models were built using the features selected by the RF algorithm. Model specification and configuration were carried out by optimization of the hyperparameters. The AD was also determined, and all compounds were found to be below the threshold of h* = 0.42, as shown in the Williams plot in Figure 3. The AD is the chemical space formed based on the descriptors of the training set compounds. The compounds in the chemical space are considered reliable for predictions, while those beyond the AD would not guarantee accurate predictions. The AD plays an important role in determining the uncertainty of the predictions of specific molecules based on how close they are to the training set compounds used to develop the model. AD is a valuable tool for the characterization of interpolation spaces based on the modeled descriptors and response functions.

**Table 2:** Statistical parameters and selected features from the developed ML models.

Method	*R* ^2^	*Q* ^2^_(LOO)_	MAE_train_	RMSE_C_	*Q* ^2^ *_F_*_1_	*Q* ^2^ *_F_*_2_	MAE_test_	RMSE_P_	Optimized hyperparameters

Random Forest	0.96	0.72	0.13	0.2	0.94	0.94	0.14	0.19	'max_depth': none, 'min_samples_leaf': 1, 'min_samples_split': 2, 'n_estimators': 80, max_features: 1.0, bootstrap: true, random_state: 0
Gradient Boost	0.99	0.77	0.06	0.08	0.82	0.81	0.2	0.34	'max_depth': 6, 'min_samples_leaf': 4, 'min_samples_split': 4, 'n_estimators': 130, max_features: none, random_state: 0
Extreme Gradient Boost	0.94	0.46	0.16	0.26	0.83	0.83	0.25	0.32	'booster': 'gbtree', 'colsample_bytree': 0.3, 'max_depth': none, 'min_child_weight': 1, 'n_estimators': 70, 'subsample': 0.5
AdaBoost	0.88	0.64	0.31	0.37	0.72	0.71	0.33	0.41	'loss': 'linear', 'n_estimators': 170, random_state: none

The applicability domain developed here is based on the features of some specific heavy metal salts, that is, CdCl_2_, ZnCl_2_, MnCl_2_, CoCl_2_, CuSO_4_, NiCl_2_, Pb(NO_3_)_2_, and SbCl_3_. The developed model should be applicable to other closely related heavy metal salts.

### Diagnosis based on SHAP value

**Figure 4:**
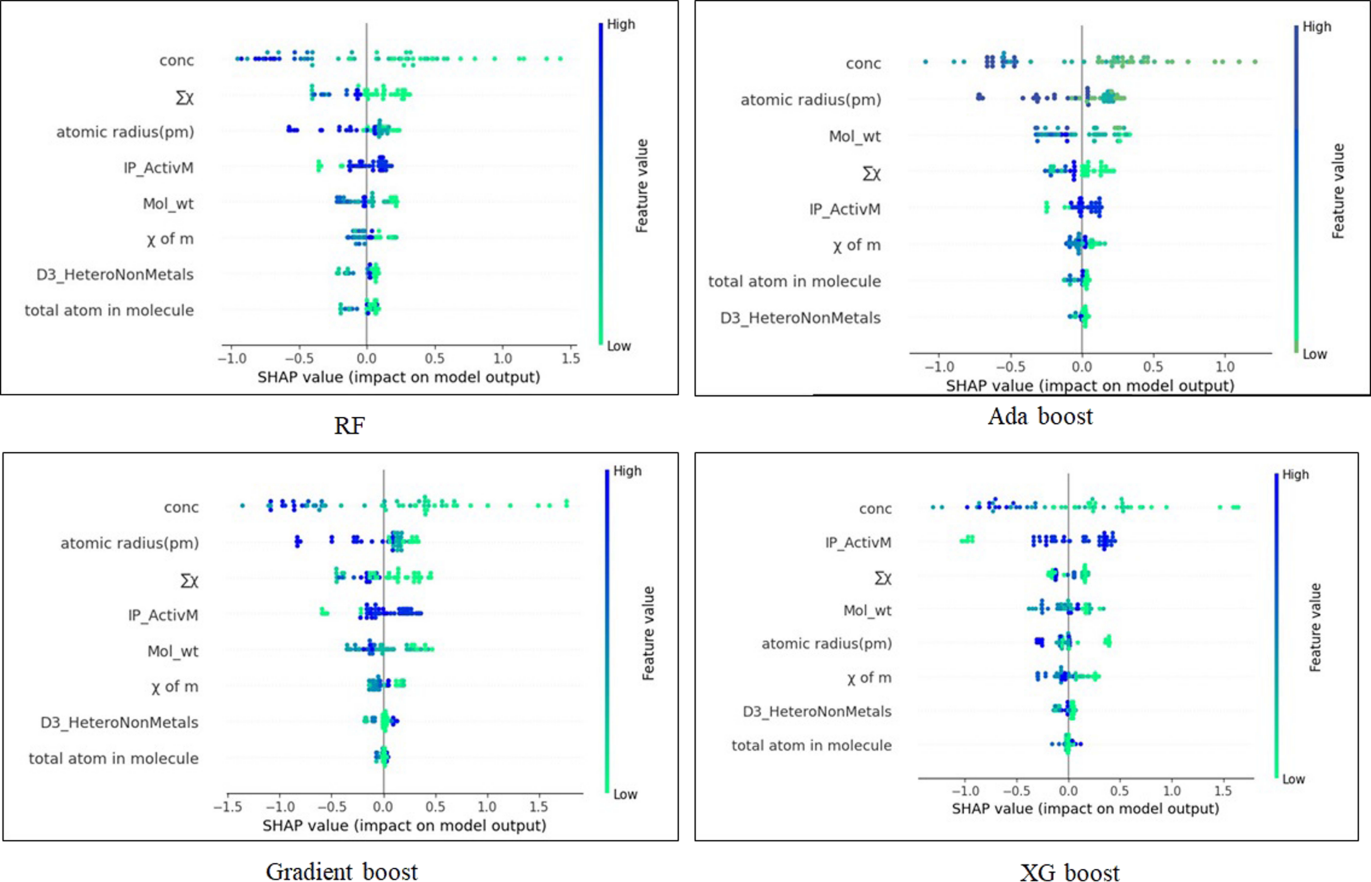
SHAP summary plot.

The goal of SHAP is to explain the prediction of an instance by computing each feature of the prediction. First, the SHAP value is used to calculate the magnitude of the contribution of each feature and then ranked to obtain the importance ranking of features. Features with large absolute Shapley values are important. Here, we have used the kernel method to calculate the SHAP values [36]. The SHAP analysis and hyperparameter tuning (max_depth: “none” min_samples_leaf, min_samples_split, n_estimators) revealed that concentration, followed by atomic radius and IP_ActivM, ranked highest among the eight features (conc, ∑χ, atomic radius, IP_ActivM, Mol_Wt, χ of metal, D3_HeteroNonMetal, and atoms in the molecule) in the RF model. The hyperparameter setting n_estimators was kept at a value of 80 for RF, while it was 130, 70, and 170 for Gradient Boost, Extreme Gradient Boost, and AdaBoost respectively. The relative importance of each descriptor for all ML algorithms can be understood using the SHAP analysis (Figure 4). The SHAP methodology identifies the features contributing most to the model prediction. We can find that the conc (concentration of the heavy metal) descriptor contributes the most to the EL algorithms. The Shapley values reflect the average marginal contribution of a feature value across all possible feature coalitions, both in terms of magnitude and direction.

### Results of model validation for all ML methods

**Figure 5:**
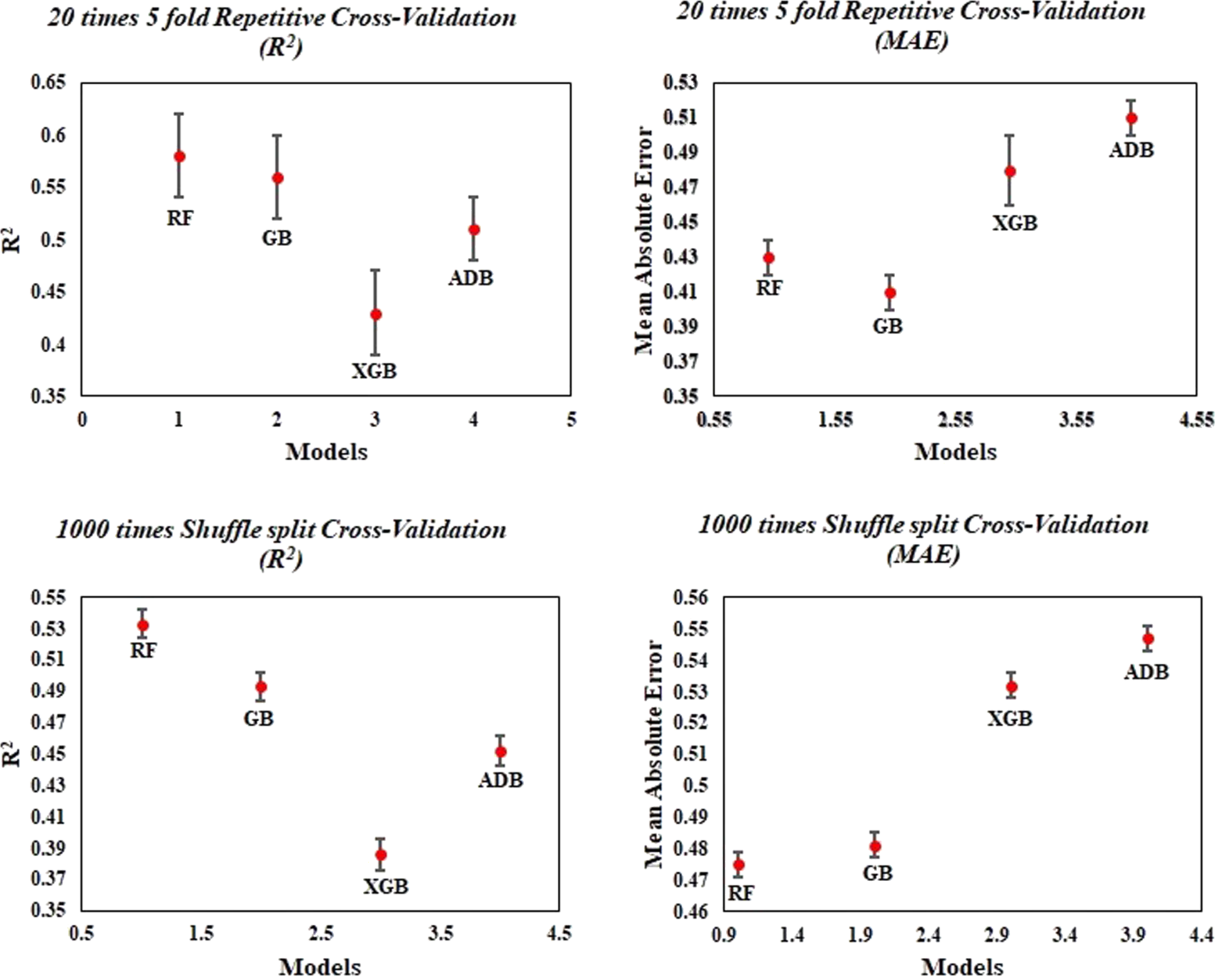
Cross-validation statistics based on 20 times fivefold repetitive CV and 1000 times shuffle split CV method (mean ± SEM).

In order to determine if heavy metals and TiO_2_ nanoparticles had any cytotoxic effects, the selected eight important periodic table-based features were used. The final models developed with RF, AdaBoost, Gradient Boost, and Extreme Gradient Boost were evaluated using MAE_train_, RMSE_train_, *R*^2^, and *Q*^2^ for the training set and MAE_test_, MSE, RMSE_test_, *Q*^2^*F*_1_, *Q*^2^*F*_2_ metrics for the test set, and the results are shown in Table 1. According to the results, the MAE_test_ (0.14) was found to be the least for the test set in the RF method, followed by AdaBoost, Gradient Boost, and XGBoost. The XGBoost gives the highest *R*^2^ (0.99) for the training set, while AdaBoost gives the lowest *R*^2^ (0.88) with the highest MAE_test_ (0.33). Cross-validation (CV) statistics were obtained based through 20 times fivefold repetitive CV along with 1000 times shuffle split CV (mean ± SEM) method. This is done to protect the model from overfitting when the data is limited. The results of the CV indicate clearly that the models do not memorize the correspondence between the descriptors since the outcome of *R*^2^ is highest and the MAE value is lowest for the RF model after the repetitive CV method. This suggests the superiority of the RF model to other models. Figure 5 presents the cross-validation statistics based on 20 times fivefold repetitive CV and 1000 times shuffle split CV on *R*^2^ and MAE for the developed ML model.

### General mechanism of toxicity

**Figure 6:**
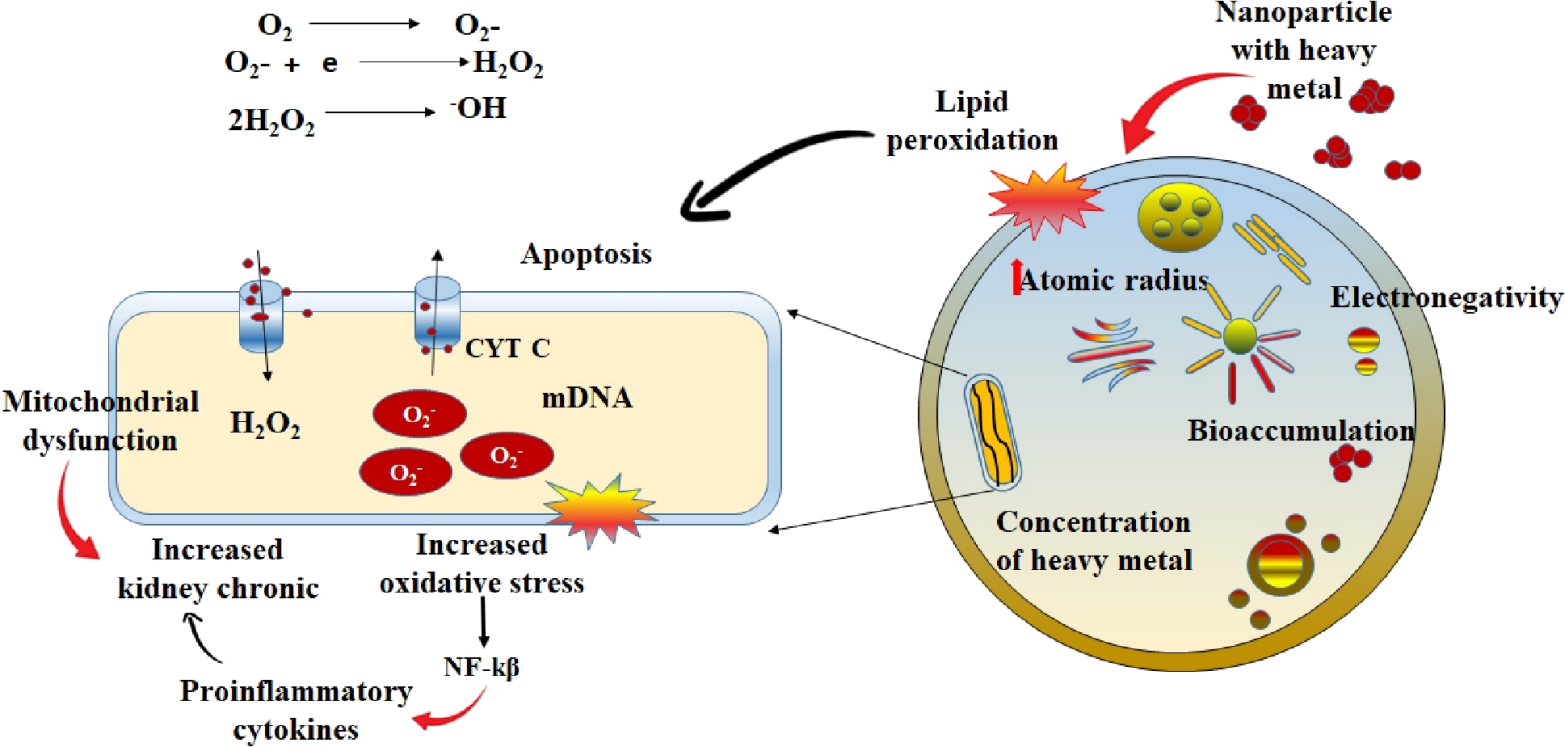
General mechanism of toxicity to HK-2 cell by heavy metals.

In the process of screening all descriptors from different ML methods, some common descriptors for heavy metals were discovered that are clear indicators of their importance regarding toxicity to HK-2 cells. We found that the concentration of the heavy metal (conc), the atomic radius of the metal, the electronegativity, and the molecular weight of the heavy metal influence the survival rate of the HK-2 cells. It was observed that conc, mol wt, atomic radius, and the total number of atoms in the molecule were of high importance in all the models. The increase of conc, mol_wt, and total atoms in a molecule is believed to increase toxicity. The toxicity of the heavy metals is also time- and dose-dependent. Among many other factors, the valence state plays an important role in toxicokinetics and toxicodynamics. Many studies have shown that an increased concentration of heavy metals is correlated with the severity of hepatotoxicity and nephrotoxicity [37]. Lead causes toxicity through an ionic mechanism followed by the generation of reactive oxygen species (ROS). Another, biomarker for ROS is lipid peroxidation [38] as free radicals cause lipid peroxidation inside the cell membrane. The catalytic properties of the metals are also responsible for an increased toxicity of manufactured nanoparticles [39] (Figure 6). Electronegativity and atomic radius influence the catalytic properties of the metal. Metal cations also catalyze the lipid peroxidation process [40] through enhancement of endocytosis and the intrinsic properties of the heavy metal. The toxicity is associated with internalization and bioaccumulation in the HK-2 cells. The increase in the concentration of heavy metals and their adsorption to nano-TiO_2_ induces toxicity by increasing the generation of ROS in the HK-2 cells.

### Comparison with the previous work

The present work describes the development of a model for heavy metals with different concentrations through simple periodic table descriptors using various ML methods. The results obtained from the ML method suggest that the models have better predictivity than the models developed previously by Sang et al. [20] as shown in Table 3. Sang et al. [20] applied the random forest algorithm and the AdaBoost algorithm for QSAR modeling using quantum mechanical descriptors. In contrast, the present study involved the random forest algorithm, the AdaBoost algorithm along with Gradient Boost and XGBoost algorithms using simple periodic table descriptors that are easy to interpret and can be calculated quickly without the involvement of expert personnel. These descriptors simplify the nanostructure property calculation and determine the nanoscale interactions without much computational intervention. The use of such descriptors saves time; the descriptors are also cost-effective and have a clear and straightforward physical meaning, which facilitates the mechanical interpretation of the QSAR models. A direct comparison was not possible due to different dataset division and descriptors but the results obtained in the present work for the RF method was superior to that of the previous work.

**Table 3:** Comparison of the current work with the previous study.

Descriptors	Method	*R* ^2^	*Q* ^2^_(LOO)_	MAE_train_	RMSE_C_	*Q* ^2^ *_F_*_1_	*Q* ^2^ *_F_*_2_	MAE_test_	RMSE_P_

periodic table-based (current study)	random forest	0.96	0.72	0.13	0.2	0.94	0.94	0.14	0.19
quantum mechanical (Sang et al.)	random forest	0.85	0.70	—	0.06	0.86	0.85	—	0.10

## Conclusion

We have performed cytotoxicity modeling of eight heavy metal compounds adsorbed on nanoscale TiO_2_ regarding HK-2 cells and explored the features responsible for the toxicity mechanism. Many studies have examined the co-exposure of metal and metalloid mixtures with heavy metals. The co-exposure may also be affected by dose variations at the biomarker level. Also, co-exposure in humans was found to lead to more profound renal damage than exposure to each of the elements alone. Hence, to elucidate the features responsible for the toxicity, in the present study, ML algorithms were applied along with periodic table descriptors for QSAR modeling. Experiment-independent periodic table descriptors produced better results than quantum chemical descriptors in previous studies. The periodic table descriptors used in QSAR models have strong theoretical guidance, which can help scientists design new entities with expected properties. As a part of the model development process, periodic table descriptors can be used in conjunction with other descriptors that are compatible with them. The periodic table descriptors are not only less computationally demanding but also independent of the size of the particles. The ML algorithm with periodic table descriptors has helped to evaluate the cell survival rate of HK-2 cells in less time and at less cost than using expensive quantum chemical descriptors and experimental descriptors. Among all algorithms, the random forest model shows the best prediction ability with *Q*^2^*_F_*_1_ = 0. 94 and MAE_test_ = 0.14 for the test set. Hence, a good feature selection method reduced the computation time required to train a model. The SHAP analysis also emphasized the most significant features contributing to the model. We have proposed also a generalized mechanism for the most impactful features generated by the model. As a result, periodic table descriptors and machine learning can be used together to decipher features of unknown compounds and predict compounds that are similar.

## Supporting Information

File 1 Detailed information regarding heavy metals at different concentrations.
